# Determinants of dental care use in patients with rare diseases: a qualitative exploration

**DOI:** 10.1186/s12903-023-03048-1

**Published:** 2023-06-22

**Authors:** Lisa Friedlander, Ariane Berdal, Valérie Cormier-Daire, Stanislas Lyonnet, Nicolas Garcelon

**Affiliations:** 1 Université Paris Cité, Laboratoire ECEVE INSERM, UMR1123, Hôpital Robert Debré, Paris, France; 2 Centre de Reference, Maladies Orales et Dentaires Rares, Hôpital Rothschild, APHP, Paris, France; 3 Filière de Santé Maladies Rares TETECOU, Malformations Rares de la tête, du cou et des dents, Hôpital Necker, Paris, France; 4 FHU DDS-Net, Dental School, Université de Paris, Paris, France; 5 Imagine Institute, Université Paris Cité, INSERM UMR 1163, Paris, France; 6 Imagine Institute, 24 boulevard du Montparnasse, Paris, 75015 France

**Keywords:** Rare disease, Quality of life, Care course, Disabilities, Orality disorders

## Abstract

**Background:**

Oral health is an inherent part of overall health as an important physiological crossroad of functions such as mastication, swallowing or phonation; and plays a central role in the life of relationships facilitating social and emotional expression.Our hypothesis was that in patients with rare diseases, access to dental care could be difficult because of the lack of professionals who know the diseases and accept to treat the patients, but also because some patients with cognitive and intellectual disabilities could not find adequate infrastructure to assist in managing their oral health.

**Methods:**

This study employed a qualitative descriptive design including semi-structured interviews using guiding themes. The transcripts were reviewed to identify key themes and interviews were performed until the data were saturated and no further themes emerged.

**Results:**

Twenty-nine patients from 7 to 24 years old were included in the study of which 15 patients had an intellectual delay. The results show that access to care is complicated more by aspects concerning intellectual disability than by the fact that the disease is rare. Oral disorders are also an obstacle to the maintenance of their oral health.

**Conclusion:**

The oral health of patients with rare diseases, can be greatly enhanced by a pooling of knowledge between health professionals in the various sectors around the patient’s care. It is essential that this becomes a focus of national public health action that promotes transdisciplinary care for the benefit of these patients.

## Background

In the European Union, a disease is considered rare when it affects less than one person in 2000. Awareness of rare diseases has increased in the EU since 2009, when the Council of the European Union asked Member States to develop plans and strategies on rare diseases [1].

Although the conditions are rare, the total number of patients affected is significant (3–4 million people in France, 27–36 million people in the EU and 25 million in the US). To date, 5,000 to 8,000 distinct rare diseases have been documented and newly discovered rare diseases (RD) are regularly reported in the literature. Most definitions seem to consider the prevalence of the disease, but sometimes other criteria apply, such as the severity of the disease, and whether it is hereditary [2].

In France, the management of rare diseases has been institutionalized through different national plans - *Plan National Maladies Rares* (PNMR). The first one, included in the law on public health policy of 9 August 2004, was implemented from 2005 to 2008. Currently, the third PNMR (2018–2022) is a continuation of the two previous ones. These plans have set up the rare disease expert centres and networks covering the entire territory to give access to care and expertise for all affected individuals. Their clinical and biological parameters are collected as shared information to pilot patient care and prevention at the national and eventually European level and provide databanks for research on rare diseases [3].

One common denominator of all patients affected by a rare disease is the relevance of their oral condition [4, 5]. Optimal oral health will limit the impact of nutritional disorders on their pathology and reduce the occurrence of microbial infection and inflammation. Sound oral health will also preserve the smile and facial form, thus maintaining function and limiting aesthetic damage and therefore preserving the individual’s self-esteem in social interactions. Rare disease patterns may include a constitutive oral phenotype or not. In these last cases, other pathophysiological traits may indirectly affect oral functionality and/or diseases such as impaired microbial defenses, motor capacities, eating or cleaning behavior. For instance, patients can have in their clinical picture an intellectual delay and an acquisition disorder, or not. All these constitutive and/or indirect oral features are important to consider analyzing in an accurate and efficient way, the oral care pathways and the resulting quality of life related to oral health [5–7]. Indeed, oral health is an inherent part of overall health as its physiological crossroads house basic functions such as mastication, swallowing or phonation, and oral health plays a central role in the life of relationships (aesthetic and social handicap, emotional feelings) and individual self-image and consideration [8].

The general hypothesis was that in patients with rare diseases, access to dental care could be difficult [1] because of the lack of professionals and expert structures in a situation to diagnose, follow and treat their disease in the necessary multi-disciplinary manner and/or [2] because some patients, such as for instance the ones with cognitive disabilities, could not find the existing adequate structures to manage their oral health.

For example: in the case of rare diseases involving oral disorders, especially in patients fed by enteral or gastrostomy methods during childhood, the practical question of the use of the oral sphere was questioned as it raises major difficulties [9, 10].

Our objective was to describe what could be the determinants of adequate access to dental care in a context of rare diseases, alongside organizational, technical, and human obstacles causing absent or inadequate management of the patients.

## Methods

### Design

This study employed a qualitative descriptive design including semi-structured interviews using guiding themes.

### Participants

In the specific context of rare diseases, it is realistic to exploit the number of patients available in a study and reach the point of saturated data than rather define the number of patients needed a priori [11].

Given the diversity and the number of diseases encountered by the study and the systematic non-overlap of the problems encountered by patients, about thirty patients, over a 6-month period, were included in the study to ensure a wide range of responses that covered the guiding themes.

Data saturation was achieved with 29 patients.

At the beginning of the planned care consultation, the practitioner and the researcher briefly but clearly explained the study and obtained the assent of the person taking part in the research, or that of his/her legal representative (Table 1).

**Table 1:** Demographic characteristics of study participants

Patient	Gender	Main Diagnosis	Age	Intellectual delay
1	M	Biliary atresia (liver transplantation)	11	Yes
2	M	Non syndromic biliary atresia	13	Yes
3	M	Methylmalonic acidemia with neonatal coma, (kidney transplantation)	20	Yes
4	M	VACTERL association, Neurogenic vesiculo-sphincter dysfunction in the context of an upper anorectal malformation associated with a filum lipoma	10	Yes
5	F	Di George syndrome: Low anorectal malformation with recto-vestibular fistula, congenital heart disease: double ventricular septal defect peri-membrane and trabecular	12	Yes
6	F	Postnatal diagnosis of double discordance with pulmonary atresia and IVC in dextrocardia, of antenatal diagnosis.Q210 interventricular communication; discordant ventriculo-auricular communication.	7	No
7	M	Biliary atresia (liver transplantation)	8	Yes
8	F	Sickle cell disease (SCD) (drepanocytosis)	15	Yes
9	M	esophageal atresia, type III associated with an omphalo mesenteric duct with common mesentery.	10	Yes
10	F	MoyaMoya disease, with multiple strokes	19	Yes
11	F	Esophageal atresia type 3	7	No
12	M	SYNGAP1 mutation	12	No
13	M	Extensive Hirschsprung’s disease - Small bowel transplantation - Chylous ascites	15	Yes
14	F	Polymicrogyria	17	Non
15	M	SATB2 Mutation	15	Non
16	F	liver-renal transplantation for methylmalonic acidemia.	18	Yes
17	M	Soto’s syndrome	15	Yes
18	M		18	No
		probable VACTERL association, partial agenesis sacral anomaly, high anorectal malformation, aortic bicuspid heart disease, right multicystic renal dysplasia with renal involution, compensatory hypertrophy of the left kidney, left vesico-ureteral reflux		
19	M	Esophageal Atresia type III	8	No
20	F	Cystic fibrosis	18	Yes
21	F	Mosaic trisomy 8	10	Yes
22	F	BPTF Mutation	10	Yes
23	M	Fibrodysplasia Ossificans Progressiva	12	No
24	F	Fibrodysplasia Ossificans Progressiva	11	No
25	M	Fibrodysplasia Ossificans Progressiva	13	No
26	M	Fibrodysplasia Ossificans Progressiva	22	No
27	M	Fibrodysplasia Ossificans Progressiva	22	No
28	F	Fibrodysplasia Ossificans Progressiva	24	No
29	F	Fibrodysplasia Ossificans Progressiva	18	No

### Ethics approval and consent to participate

The study was approved by the Ethics committee for the protection of individual COMITÉ DE PROTECTION DES PERSONNES SUD-OUEST ET OUTRE MER III (ref APHP210890/IDRCB 2021-A00429-32). In France, ethics committees are randomly assigned throughout the national territory without any notion of the authors’ affiliation.

All the patients and their parents (in case of minor’s or patient’s or mental disabilities) gave written informed consent for participation in the study and the publication of the study results:

Patients were selected in coordination with the leader of each rare disease expertise centre in the Necker Hospital (Paris, France) respectively concerned during a usual consultation at the hospital and were initially informed orally about the study. When they were interested (patients and/or their parents), an information note was given to them to explain everything and to allow them to give the most informed consent possible.

For minor patients, the parents, and their children each received an appropriate information note and the parents signed a consent document.

Adult patients who are under the guardianship (legal document) of their parent because of mental disability or medical situation, the parents signed a consent document and received the information note.

Patients of legal age who are not covered by the abovementioned, received the information note and signed the consent document.

All methods were performed in accordance with the relevant guidelines and regulations.

### Eligibility criteria

Patients were selected in coordination with the leader of each rare disease expertise centre in the Necker Hospital (Paris, France), respectively concerned.

Inclusion criteria were:

- Patients with a rare disease (diagnosis of rare disease selected by the hospital medical teams).
- Patients seen between 1.1.2017 and 1.1.2020 in the concerned Rare Disease Expertise Center.
- Patients aged 6 years and over.
- Patients who are beneficiaries of one of the social security schemes in France (general scheme or special schemes).
- Patients seen at least once in their life in the medical genetics department of the Necker Hospital ((Paris, France) because of their rare disease.

Non-inclusion criteria were:

- Patients not resident in France.
- Patients who do not speak French.

### Procedure

A semi-structured interview guide was developed by the research group and discussed with researchers and clinicians beforehand to ensure that it had enough rigor to achieve credible data collection.

The interviews all start on the disease itself and the overall care pathway. We gradually move into the topic of oral health by addressing the themes of access to care and past experiences. Most of the time, the topics intertwine, with patients and parents expressing their feelings about the overall care and dental care. It is in the analysis of the themes and sub-themes that the ideas are classified.

Some patients have a significant intellectual delay, and, in this case, it was the parents who answered the interview. For the patients who did not have an intellectual delay, they answered the interview, certainly sometimes helped by their parents, but they had full voice to express absolutely everything they felt.

### Interview

#### Data collection

Patients who met the inclusion criteria were recruited from the expertise centres or during their usual consultation, with their non-objection and that of their legal guardians if they were minors.

Face-to-face, semi-structured lasting interviews were held by the first author (L.F), trained in qualitative sociological and anthropological interview methods. All interviews took place in *Necker Enfants Malades Hospital*, (Paris, France), in a dedicated room after a standard consultation. We used an interview guide, structured by the following six themes: “Access to dental care “, “Oral health related quality of life,” “Orality disorders”, “Renouncement to dental care” and “School integration and daily life.”

During these interviews, themes were discussed with the patients who were encouraged to talk freely.

Interviews with duration between 30 and 60 min were conducted and recorded in the period of May 2021 until December 2021. Transcripts and other sensitive data were stored at Research Clinical Unit in *Necker Enfants Malades Hospital*, (Paris, France).

#### Qualitative thematic analysis

For the analysis of the qualitative data, all the recorded interviews were manually transcribed and then analyzed using a thematic data analysis software (NVIVO 12 on Windows). The thematic analysis of the questionnaires was carried out according to the following process and we are inspired in this analysis by the work of Vaismoradi, M. and all, who have been of great help to us and whom we thank [11–13]:

1. Key themes, or big ideas, were identified by reading and re-reading the interview transcripts.
2. Phrases or blocks of words that correspond directly to the research question were highlighted.
3. These phrases were categorized so that they can be grouped together thematically.
4. These categories gave rise to sub-themes which were then examined and analyzed.

The transcripts were reviewed to identify key themes and coded by a single researcher. Interviews were performed until the data were saturated and no further themes emerged, as is the current standard for qualitative data analysis in health settings.

10% of the interviews (randomly chosen) were analyzed by a second coder to ensure consistency and quality assurance of the data.

Key themes were organized in a table and those mentioned by three or more patients were considered common.

Key common themes were converted into questions and grouped into general themes.

#### Data credibility and rigor

Data credibility was established through a triangulation strategy, which uses a combination of specialized teams to review and evaluate the results. In addition to semi-structured interviews, data credibility was ensured by note taking during the interviews. Data were verified by both peers outside the study and by research team members. The main findings were presented to some of the participants and their opinions, collected. In addition, the results were evaluated and verified on several occasions by supervisors [14, 15].

Furthermore, all the authors of this article are trained in social epidemiology using a mixed method (quantitative and qualitative).

## Results

### Sample Analysis

In this study, 29 patients between the ages of 7 and 24 years of age were interviewed. Parents were present during the interviews when children or older patients wished.

The pathologies, age, gender, and presence or absence of intellectual delay of the patients are described in Table 1.

The pathologies found in the sample are for the most part syndromic, with the first signs appearing in antenatal or early childhood. The pathologies are here digestive, pulmonary, cardiac, renal, or neurodevelopmental. There are no rare diseases in the study sample that exclusively affect the orofacial sphere.

Of these 29 patients, 15 patients had an intellectual delay.

### The exploration of themes and verbatim

As envisaged in the preparation of the interview guide, four main themes were identified as keys: dental care course, mental disabilities, and orality disorders, and finally social impact.

All analyses were carried out using exact transcripts of the interviews, whether the verbatims were those of the patients or of the patients’ relatives when they were unable to express themselves.

**Dental care course** (concepts mentioned in all the 29 interviews, with 80 occurrences).

In our study, patients affected by a rare disease with an either constitutive or indirect oral phenotype had difficulty to find a practitioner who would face the overall disease context and manage their oral health. They described difficulties to get an appointment and when they found a dentist, they expressed the feeling of being rejected and referred to another-one. Parents describe the feeling of not being listened to, even though they feel that they sometimes know their child’s illness better than the dentist. In our sample, we recruited patients in extreme situations with technically very difficult treatments.

In our sample, this was exemplified in two contexts where the oral dysfunction is major: patients with fibrodysplasia ossificans progressiva (FOP), and the Di George syndrome. For FOP, the patients can have - and this was the case for 4 of the 7 patients in the study- a total ankylosis of the temporo-mandibular joints because of heterotopic ossification. This ankylosis makes almost impossible for them to open their mouths (opening of less than 1 cm) and complicates dental care. It is also known that these bone flare-ups can be caused by muscle trauma during dental care. Patients are therefore very apprehensive about dental care.

These parents of a child with FOP described a rather chaotic oral health care journey:

*“The first dentist he saw was a friend of our pediatrician. He lived far away from us, but we needed someone we could trust, because the mouth is an extremely sensitive area for FOP Patients, so the dentist really had to understand that he shouldn’t be afraid of a FOP patient, but he shouldn’t open his mouth too much.“*

*“I basically write: FOP, etc., thinking a little bit like you stupidly: she’s going to ask about it. She arrived, didn’t even look at me and I was the one who had to say to her: “Wait, Madam, we’re going to stop, I’m going to explain the situation to you”, and so, that was still disturbing.*

*“But it’s true that we arrive with a bit of annoyance because I had also taken the trouble to fill out her paperwork, she didn’t even look at it, it’s obvious.*

The theme of oral care and everything that affects the oral cavity appeared to all the patients and parents interviewed as a major and integral part of the overall care.

Parents did report that when it was possible to have the child’s teeth treated in the same place as the overall management of the disease, the care pathways were extremely simplified. Professionals were able to work in better communication with each other and improve management.:

*“Last time at the dentist, I felt like they’re afraid to touch it. They’re hesitant. Like this one, Dr. xx she takes children, I know that, but in relation to him, she told me no and sent me to another one.*

*“Already to get an appointment, it was very difficult to find someone who could do dental care”*.

Parents describe what they see as a lack of knowledge on the part of dentists, and sometimes what they interpret as fear of treating their children:

*“The dentist we had taken near our home, he didn’t want to do extractions that were a little complicated like that, so he told us to do it somewhere else. We started by going to a dental clinic. When we saw the estimates, we said to ourselves: “He doesn’t care about us”.*

*“Yes, she didn’t know, you could tell.“*

*“That’s also the flaw when you’re a parent, is that you have your nose in the grindstone so much, I realize that with teachers, when we explain things, it seems so easy. It’s like, I think, with dentists, you don’t want to stretch, you don’t want to spread it too far, you don’t want to… and in fact, we don’t necessarily think to say it.“*

*“Diane, we did what we did for her brother. When she was very young, we took her to the dentist once a year for check-ups and the first few times, the dentist just looked at her and patted her teeth to reassure her. Then she got used to going to the dentist. You weren’t afraid.“*

*“Because at 18 months, the family dentist didn’t know what was wrong with her, she was losing enamel on the face of the teeth, and it was getting very white and not ivory at all.“*

**Mental disabilities** (concepts mentioned in 15 interviews, with 45 occurrences).

The parents who were interviewed and had a child with an intellectual disability emphasized the difficulty of access to dental care for their children. Parents reported inappropriate psychological behavior regarding the whole family with apparently untrained and stressed dentist regarding their children.

*“It’s true that disability is scary already, and I think they’re not trained.“*

They may report comments that they feel have hurt them as parents and parents well describe the lack of therapeutic education and assistance in implementing effective dental hygiene protocols.

*“The last time we went there, 1 month ago, I had the reflection of the dentist who said to me: ‘it is up to you to do the oral hygiene of your daughter’. But it’s not easy with a child with a disability to brush her teeth, it’s not at all obvious. And when I explained that they got angry at me. They said, “Yes, you are responsible for your child. Do you realize, after 1 year, she will have cavities again”, I said I knew it very well.“ “It’s true that disability is already scary, and I think they are not trained.“*

*“In private practice, anyway, I think there’s the number to go behind. If they spend three quarters of an hour doing a treatment and they don’t succeed, it’s not profitable for them. I’m not saying this well, but afterwards, they have expenses, which is also understandable. So they don’t necessarily have the time or the desire, or the diplomacy, to take care of children with disabilities.*

*“She was screaming so much that the dentist said that She was scaring all his clients.“*

**Orality disorders** (concepts mentioned in 12 interviews, with 40 occurrences).

Many oral problems were mentioned by parents. These difficulties with the oral sphere concerned all patient functions: feeding, cleaning, speaking. In this context, oral care was complicated, even in children who did not have intellectual delays.

*“She doesn’t like it when you put something in her mouth. Better now, but a year or two ago, she didn’t like anything in her mouth. Even the toothbrush was difficult. I was the one who had to brush, but that was hard too. She would gag every time you went in the back a little bit. It was very, very, very difficult.*

*“Since baby, she always had trouble putting things in her mouth. Foods. Sometimes foods that were a little greasy or whatever, it was hard. It was always a heartache.*

*“It’s always been hard on the food part since she was a little girl. Even now. It’s hard to identify something, to say she doesn’t like this texture, or she doesn’t like this consistency, etc., because it’s kind of like it’s diffuse and it’s very hard to identify the structuring. I say anything: she doesn’t like orange and she won’t eat carrots. It evolves and we’re not sure why.“*

*“She had hyper nausea. Chewing was very complicated, even now she can chew, but it shouldn’t be too…”*.

*“From the moment it was to eat, she took her pacifier, I had a pacifier where we could put milk, medicine and she spit out the pacifier. It wasn’t the same function anymore; we had changed the function of the pacifier.“*

*“Care was impossible because you had to get into the mouth.“*

*“We were behind in getting good oral hygiene, because he had sensory dysorality syndrome.“*

*“As a result, nothing would fit in his mouth. He had his sensitivity threshold at the entrance to his mouth. In fact, he had bitten me once, so he never teethed on things.*

*“He was gagging. The toothbrush, it wasn’t even worth it.“*

*“Chewing is not at its best. If he can swallow whole, he does. I have to remind him to chew.“*

*“To chew, it’s very complicated.“*

*“He had periods when he could eat, because we weren’t sure how to do it, whether to eat, not eat, infuse, enteral, eat a little bit. He was in occlusion very regularly and as a result, this really altered his orality in the sense that he did not develop his feeding as a toddler, where you gradually integrate milk, certain foods, textures, etc. In relation to that, he developed food phobias.“*

*“It was intrusive, and then because he wasn’t eating, it was intrusive to put in his mouth. It was complicated, I would say that he hasn’t been in the habit for very long, and you still have to be behind it, because it’s not something natural.*

*“Yes. The mouth noises, she can’t stand, she doesn’t eat with us anymore. She already can’t stand herself, hearing her noises.“*

*“No, I think it’s subsiding. I think it’s people who are learning, who are less excited. Afterwards, it’s also Alexandre’s character that wants that, he’s a dynamic boy, who likes it when it pulsates, who likes it when it goes fast. Afterwards, I think that as we grow up we learn to settle down and the FOP forces us to settle down even more.*

**Social impact** (concepts mentioned in 10 interviews, with 30 occurrences).

The patients and their parents did not return to the theme of social and school integration as much as to the themes of the dental care pathway. Nevertheless, the notions of the look of others, of appearance, of others were raised, especially by the older patients.

*“My teeth, I don’t have a nice smile like some of my friends. They have beautiful teeth, a beautiful smile, and I am too ashamed to smile in front of others. To laugh, I put my hand in front of my mouth, it has become a huge complex.*

*“That’s also the flaw when you’re a parent, it’s that you have your nose in the grindstone so much, I realize that with the teachers, when we explain things, it seems so easy. It’s like, I think, with dentists, you don’t want to stretch, you don’t want to spread it too far, you don’t want to… and in fact, we don’t necessarily think to say it.“*

*“Because when you’re talking, people… sometimes you don’t realize, but sometimes there are people, they don’t look you in the eye, but they look at your lips or your teeth, how they look. And then, when you’re done talking, they’ll say: “But why are your teeth like that?*

*“They are friends with me because of who I am and not because of what they see. They are friends with me for who I am, not for what they see. For my character, for my good mood, not for my disabilities, because I can’t go to Paris and I can’t take the metro. They know that, if they go to Paris, they won’t offer it to me, because it will be a direct “no”. But for example, if we go somewhere to eat, we go by bus, we’ve already thought about everything, we’re not going to do anything too complicated.“*

## Discussion

### Discussion of results

The results of this study can be summarized in three major several points.

First point, in our study, patients with a rare disease including a behavioral disorder (neurodevelopmental disorder, delayed acquisition) have a lack of access to dental care in appropriate conditions such as sedation and a team accustomed to this care. The oral management of patients with cognitive and mental disabilities goes far beyond the notion of a rare disease. Barriers to care are permanent for patients and their families with care structures not always adapted to receive these patients. For these patients, a therapeutic arsenal of sedation, whether conscious (hypnosis, nitrous oxide) or general anesthesia, is necessary. This requires adapted care premises, adequate equipment, trained professionals, and care teams that are often larger than for patients without these disabilities [16, 17].

In this type of theme, where the feelings of the patients and their parents are major elements of understanding the problems, qualitative research provides a detailed understanding of patients’ perspectives and expectations and can be an essential first step in the development of future patient-centered measures. Qualitative research and its methods provide opportunities for a systemic and holistic understanding of the difficulties faced by patients. Qualitative methods are increasingly used in the medical literature to understand the issues of patients’ daily lives and care pathways [16, 18]. It seems interesting, and it is an originality of this study, to consider all the rare diseases as a whole and to give a global approach which is opposed to the usual literature which is interested in the diagnostic and therapeutic elements in each disease.

This creates in these patients a renunciation of dental care, an oral health judged degraded by the parents and resentment expressed by the parents as well.

The parents describe the very chaotic dental care pathway for these patients with difficult but often indispensable access to general anesthesia for care.

In addition, in our study, the difficulties of oral management of patients with orality disorders, often caused by parenteral nutrition or gastrostomy, are an important point raised by the parents. Complications related to feeding, food choice, oral hygiene, and acceptance of oral care, even in children without neurodevelopmental delays, appear to be major.

The second main point is that, overall, patients describe a lack of knowledge of their pathologies by oral health professionals. The complexity of management for professionals in terms of oral health is multiple. On the one hand, the rarity of the disease requires the patient to organize a care network that is often complex in terms of access to competent professionals trained in the management of this disease, and on the other hand, professionals who are not trained in the phenotypic specificity of this disease may have limitations in their management and direct patients in unsatisfactory therapeutic directions [19, 20] .

These difficulties appear clearly in patients with oral disorders who have been parenterally fed, e.g., with biliary atresia. These patients have an altered relationship to all their oral sphere and inappropriate management will necessarily have consequences on the oral quality of life of patients and their general quality of life [21, 22].

The third important result of this study concerns the difficulties of access to adequate dental care for patients with rare diseases with a significant oral and dental component. The difficulties are more technical for the professionals than behavioral. For example, concerning patients with *fibrodysplasia ossificans progressiva*, an ultra-rare bone disease, where heterotopic bone growths can cause ankylosis of the temporo-mandibular joints, in case of dental care, all expressed fears about dental care and a great ignorance of their pathology by the dental surgeons [23–25]. These elements caused a delay in access to oral health care and a therapeutic wandering until the adequate professionals were found.

### Strengths

Few studies have investigated determinants of dental care use of these patients, both children and adults. These studies use quantitative methods, the main measure being standardized questionnaires [19, 26]. These questionnaires, although very useful for guiding and highlighting certain aspects of the impact of the disease on oral quality of life, are nevertheless limited in their precision concerning the feelings and experiences expressed by the patients. For these diseases, there is no recent data in the literature from qualitative work based on interviews or focus groups.

All the patients included in the study are followed in a hospital very specialized in the management of rare diseases with many centers of expertise. For most of them, the care network is nowadays rather well established, even in the field of oral health. We can consider this point as a bias in the analysis of the study, however, many of the patients’ verbatims concern the difficulties they may have had and still have in accessing adequate dental care, which shows that even if they are very well taken care of for their pathology in a global way, oral health remains an often very chaotic point.

### Limitations

Necker Hospital in Paris, where this study was conducted, receives patients with very rare diseases such as Fibrodysplasia ossificans progressiva. We wanted to include these patients whose dental care pathway is quite singular given the specificity of this pathology (TMJ ankylosis can be caused by iatrogenic dental care). We are fully aware that, given the rarity of this disease but also the fact that teams very specialized in this pathology are present on site, we have a much higher recruitment than what we could have in the rest of France. This could be considered as a selection bias. Nevertheless, the observations we make about the complex oral care pathways for patients with mental disabilities, much more than the notion of a rare disease itself, depend on the territorial coverage of specialized services to receive this type of patient, potential geographical difficulties, and the possibility of accessing care under general anesthesia.

We can see from this study, and the literature has already shown it in many aspects, that the “rarity” of a disease is not the most difficult point regarding the consideration of patients’ oral health.

However, it appears that one of the levers for improving care paths must be through:

- A better knowledge of the oral care of patients with rare diseases.
- A real consideration and reflection on the oral care of patients with neurodevelopmental and intellectual delays whose access to care is clearly lacking.
- The promotion of oral health in the expertise center network not directly linked to the TETECOU network (TETECOU network” is a network of French centers of expertise taking care of rare diseases of the head, neck, and teeth): prevention, therapeutic education, awareness of professionals.
- Improvement of oral health care pathways: relationship between the TETECOU network and other rare disease health networks.
- To create new, inter rare disease disciplinary consultations (inter-channels): thanks to the collaboration between the different rare disease health channels, clinical research protocols can be developed as close as possible to the needs of patients: joint consultation between dentistry and other specialties, evaluation of oral rehabilitation for patients whose oral needs have not been considered until now.

In France, and in some European countries, “national rare disease plans” follow one another and offer more and more territorial networking, centers of expertise and access to diagnosis and care for patients with rare diseases. As far as oral health is concerned, the stakes remain high as regards the most appropriate care for the different vulnerabilities of patients. It seems essential to enhance the care of patients with disabilities by improving the training of professionals, facilitating the reception of patients, and offering financial incentives to professionals. Inclusion efforts must be global and involve, to a large extent, access to care worthy of the rest of the health system.

Through this study, we hope to contribute to the coordination of different care networks on a national scale to significantly improve patients’ daily lives. The identification of psychosocial repercussions of their disability should lead to a broadening of current care with the involvement of social workers and psychologists, but above all to the institutional organization of financial support for oral rehabilitation.

## Conclusion

This study exceeded its objectives in many ways. We thought that we should once again insist on the “rare” side of the diseases concerned by our sample. We were almost surprised, even if we knew the existence of difficulties, the more than chaotic course of the care of patients with cognitive disabilities. It seems necessary to be able to promote oral health for all, in the best conditions, even when these are costly: sedations of all kinds, long sessions, general anesthesia, assistance by trained professionals (psychologists, speech therapists). Oral health care cannot be another reason for therapeutic wandering for these patients who have often already experienced diagnostic wandering for their pathology.

## Data Availability

The datasets during and/or analyzed during the current study are available from the corresponding author on a reasonable request.
